# Alteration of corneal biomechanical properties in patients with dry eye disease

**DOI:** 10.1371/journal.pone.0254442

**Published:** 2021-07-12

**Authors:** Vannarut Satitpitakul, Parichart Taweekitikul, Vilavun Puangsricharern, Ngamjit Kasetsuwan, Usanee Reinprayoon, Thanachaporn Kittipibul

**Affiliations:** Center of Excellence for Cornea and Stem Cell Transplantation, Department of Ophthalmology, Faculty of Medicine, Chulalongkorn University and King Chulalongkorn Memorial Hospital, Thai Red Cross Society, Bangkok, Thailand; Boston University School of Medicine, UNITED STATES

## Abstract

**Purpose:**

To evaluate the association between symptoms and signs of dry eye diseases (DED) with corneal biomechanical parameters.

**Methods:**

This cross-sectional study enrolled 81 participants without history of ocular hypertension, glaucoma, keratoconus, corneal edema, contact lens use, diabetes, and ocular surgery. All participants were evaluated for symptoms and signs of DED using OSDI questionnaire, tear film break-up time (TBUT), conjunctival and corneal staining (NEI grading) and Schirmer test. Corneal biomechanical parameters were obtained using Corvis ST. Mixed-effects linear regression analysis was used to determine the association between symptoms and signs of DED with corneal biomechanical parameters. Difference in corneal biomechanical parameter between participants with low (Schirmer value ≤10 mm; LT group) and normal (Schirmer value >10mm; NT group) tear production was analyzed using ANCOVA test.

**Results:**

The median OSDI scores, TBUT, conjunctival and corneal staining scores as well as Schirmer test were 13±16.5 (range; 0–77), 5.3±4.2 seconds (range; 1.3–11), 0±1 (range; 0–4), 0±2 (ranges; 0–9) and 16±14 mm (range; 0–45) respectively. Regression analysis adjusted with participants’ refraction, intraocular pressure, and central corneal thickness showed that OSDI had a negative association with highest concavity radius (P = 0.02). The association between DED signs and corneal biomechanical parameters were found between conjunctival staining scores with second applanation velocity (A2V, P = 0.04), corneal staining scores with second applanation length (A2L, P = 0.01), Schirmer test with first applanation time (A1T, P = 0.04) and first applanation velocity (P = 0.01). In subgroup analysis, there was no difference in corneal biomechanical parameters between participants with low and normal tear production (P>0.05). The associations were found between OSDI with time to highest concavity (P<0.01) and highest displacement of corneal apex (HC-DA, P = 0.04), conjunctival staining scores with A2L (P = 0.01) and A2V (P<0.01) in LT group, and Schirmer test with A1T (P = 0.02) and HC-DA (P = 0.03), corneal staining scores with A2L (P<0.01) in NT group.

**Conclusions:**

According to in vivo observation with Corvis ST, patients with DED showed more compliant corneas. The increase in dry eye severity was associated with the worsening of corneal biomechanics in both patients with low and normal tear production.

## Introduction

Dry eye disease (DED), which is among the most frequently encountered ocular disease, is a multifactorial disease that affects both ocular surface and tear film layer. Patients with DED usually presented with eye irritation, photosensitivity, and blurred vision which have an impact on both quality of life and quality of vision [1]. Previous studies using in vivo confocal microscopy have shown that DED has a significant effect on cornea at the cellular level. The changes in corneal epithelium, corneal nerves, corneal stroma and corneal endothelium have been observed. In addition, the corneal structural change was correlated with dry eye severity [2].

Corneal biomechanics includes elastic and viscoelastic properties, which are the capacity of cornea to reversibly deform under stress [3]. The biomechanical properties of cornea depend on the patterns of fiber organization and constitution in each layer of the cornea [4]. Various alterations of cornea in patients with DED have been demonstrated, including decreased corneal superficial epithelial cell density [5], increased corneal keratocyte density [6], increased inflammatory dendritic cells [7], decreased subbasal nerve plexus number, increased beadings and tortuosity of corneal nerve [8, 9], and decreased endothelial cell density [10, 11]. Moreover, ocular inflammation, which is the main pathogenesis of DED, could lead to stromal change and weakening of the corneal tissue [12, 13]. Therefore, we hypothesized that DED has an impact on corneal biomechanics and it is also interesting to illustrate whether corneal biomechanical alteration associates with the disease severity or not.

The purpose of this study is to evaluate the association between symptoms and signs of dry eye diseases with corneal biomechanical parameters.

## Materials and methods

In this cross-sectional study, 81 participants were recruited from Comprehensive Geriatric Clinic, King Chulalongkorn Memorial Hospital, Bangkok, Thailand. An institutional review board in the Faculty of Medicine, Chulalongkorn University approved the protocol of this study. The study adhered to the tenets of the Declaration of Helsinki. Written informed consent was obtained from each participant.

Consecutive participants were enrolled. Participants with history of contact lens use, ocular hypertension, glaucoma, keratoconus, corneal edema, corneal dystrophy, any ocular surgeries, corneal cross-linking treatment, and diabetes mellitus were excluded.

All participants were evaluated for symptoms of dry eye disease using the Ocular Surface Disease Index (OSDI) questionnaire. Best-corrected visual acuity, manifest refraction, intraocular pressure (IOP) and signs of DED including tear film break-up time (TBUT), conjunctival and corneal staining scores using NEI grading system and Schirmer test with anesthesia were consecutively done by trained corneal specialists.

**Fig 1:**
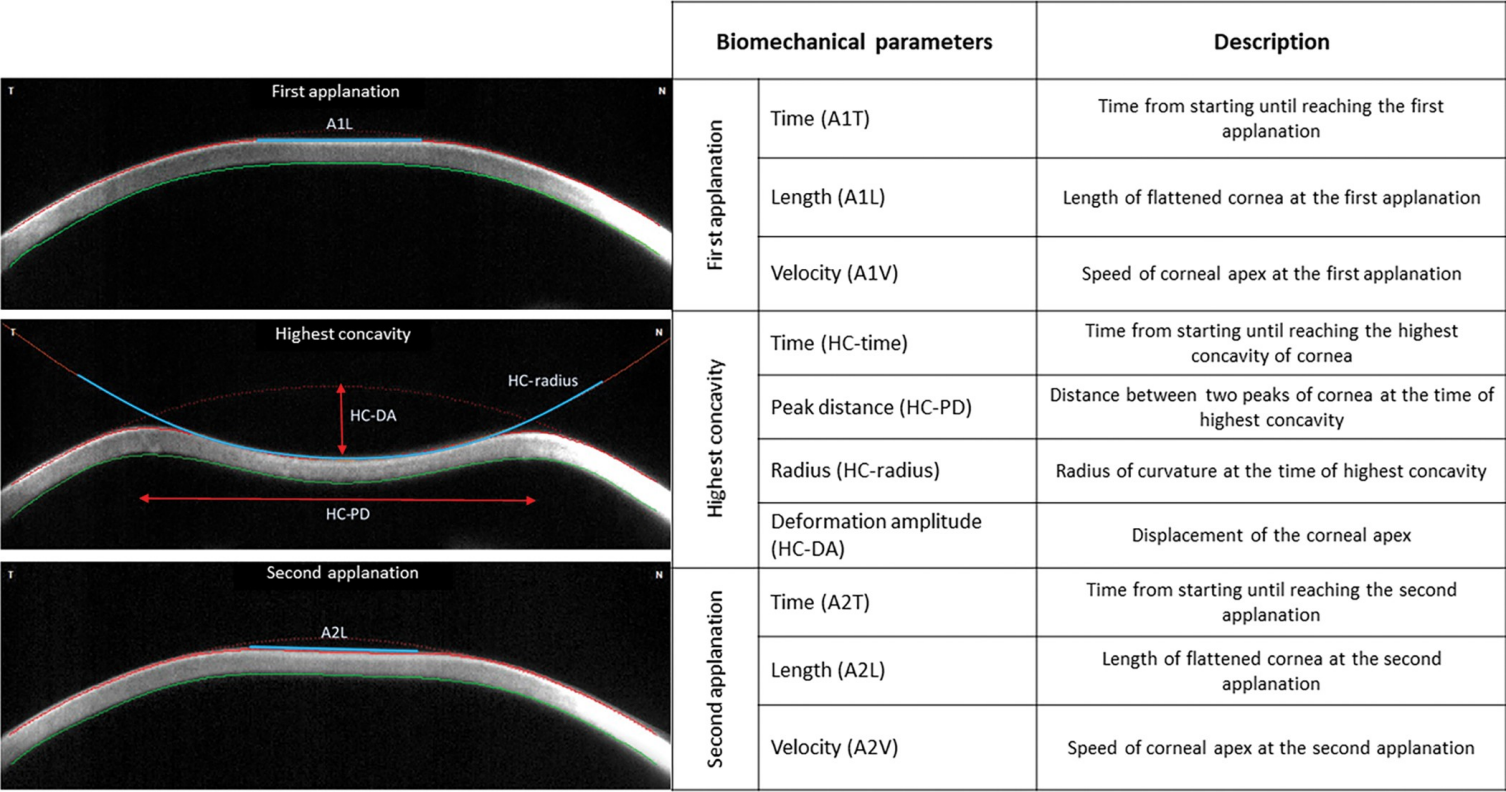
Corneal biomechanical parameters evaluated by Corvis ST.

Corneal biomechanical properties were then evaluated using Corneal Visualization Scheimpflug Technology (Corvis ST, Oculus, Wetzlar, Germany) by a single masked investigator. After an air impulse, Scheimpflug camera recorded images of the first applanation, the highest concavity of the cornea, and the second applanation, respectively. Ten biomechanical parameters were recorded (Fig 1)

The data from only one eye from each participant was used for analysis. The data from right eyes was first analyzed. If the right eye was excluded, the data from the left eye was used.

The statistical analysis was performed using Stata/IC for Windows (version 14.1, Stata Corp). The distribution of the data was tested by means of the Shapiro-Wilk test. Data with non-normal distribution were presented as median and interquartile range (IQR), and data with normal distribution were presented as mean and standard deviation (SD). Mixed-effects linear regression analyses adjusted for participants’ spherical equivalent refraction (SE), IOP, and central corneal thickness (CCT) were used to determine the association between symptoms and signs of DED with corneal biomechanical parameters in all participants. To further evaluate the effect of tear production on corneal biomechanics, the data were classified into low tear production (LT) and normal tear production (NT) groups. The LT group included participants with Schirmer value of 10 mm or less and the NT group included participants with Schirmer value more than 10 mm. ANCOVA test was used to compare corneal biomechanical parameters between groups after adjusting for SE, IOP and CCT. Mixed-effect linear regression analyses was also used to determine the association between symptoms and signs of DED with the adjusted corneal biomechanical parameters in each group. P-values of less than 0.05 were considered as a statistical significance.

## Results and discussion

This study included 81 participants who had a mean age of 66 ±3.4 years (range, 60–77 years), comprising 53 females and 28 males. Twenty-eight (34.6%), 22 (27.2%), 1 (1.2%) and 1 (1.2%) participants respectively had dyslipidemia, hypertension, rheumatoid arthritis, and history of Steven Johnson syndrome. None of the participants had allergic conjunctivitis. There were no topical eye medications used by any participants except for 39 (48.75%) participants, who were using artificial tears.

Median OSDI score was 12.50 (IQR 4.55, 20.45) and median TBUT was 5.33 seconds (IQR 3.67, 7.67 seconds). Median conjunctival and corneal staining scores was 0 (IQR 0, 2) and 0 (IQR 0, 1) respectively. Median Schirmer test was 16 mm (IQR 9, 23 mm).

Adjusting with SE, IOP, and CCT, OSDI scores showed significant negative association with HC-radius (P = 0.02). There were significant positive associations between Schirmer test and A1T (P = 0.04), and corneal staining scores with A2L (P = 0.01). Significant negative associations were found between conjunctival staining scores with A2V (P = 0.04) and between Schirmer test with A1V (P = 0.01). There were no associations between OSDI score, TBUT, corneal and conjunctival staining scores and Schirmer test with other corneal biomechanical parameters (Table 1).

**Table 1:** The associations between symptoms and signs of dry eye disease and corneal biomechanical parameters of all participants.

Corneal biomechanical parameters		Symptoms and signs of dry eye disease^a^				
		OSDI scores	Tear film break-up time	Conjunctival staining scores	Corneal staining scores	Schirmer test
First applanation time (A1T)	Coefficient ±SE	-0.001 ±0.002	0.019 ±0.011	-0.005 ±0.013	-0.003 ±0.028	0.005 ±0.003
	P value	0.73	0.07	0.72	0.91	**0.04**^**b**^
Second applanation time (A2T)	Coefficient ±SE	-0.001 ±0.003	-0.006 ±0.015	0.010 ±0.018	-0.013 ±0.039	-0.005 ±0.004
	P value	0.72	0.69	0.60	0.74	0.18
First applanation length (A1L)	Coefficient ±SE	0.001 ±0.003	-0.001 ±0.015	0.029 ±0.017	0.024 ±0.037	0.003 ±0.003
	P value	0.84	0.92	0.09	0.51	0.42
Second applanation length (A2L)	Coefficient ±SE	0.002 ±0.003	0.002 ±0.015	-0.015 ±0.018	0.097 ±0.037	0.006 ±0.004
	P value	0.39	0.90	0.40	**0.01**^**b**^	0.11
First applanation velocity (A1V)	Coefficient ±SE	0.000 ±0.000	-0.001 ±0.001	0.000 ±0.001	0.000 ±0.002	0.000 ±0.000
	P value	0.89	0.19	0.97	0.89	**0.01**^**b**^
Second applanation velocity (A2V)	Coefficient ±SE	0.000 ±0.000	0.000 ±0.001	-0.003 ±0.001	0.004 ±0.003	0.000 ±0.000
	P value	>0.99	0.99	**0.04**^**b**^	0.23	0.31
Highest concavity time (HC-time)	Coefficient ±SE	0.007 ±0.004	0.025 ±0.021	-0.029 ±0.025	-0.045 ±0.053	0.006 ±0.005
	P value	0.07	0.24	0.26	0.40	0.20
Highest concavity peak distance (HC-PD)	Coefficient ±SE	-0.002 ±0.002	-0.001 ±0.010	0.023 ±0.012	0.010 ±0.026	-0.003 ±0.002
	P value	0.28	0.93	0.06	0.70	0.14
Highest concavity radius (HC-radius)	Coefficient ±SE	-0.014 ±0.006	0.038 ±0.034	0.021 ±0.041	0.030 ±0.086	-0.002 ±0.008
	P value	**0.02**^**b**^	0.27	0.60	0.72	0.81
Highest concavity deformation amplitude (HC-DA)	Coefficient ±SE	0.001 ±0.001	-0.004 ±0.004	0.005 ±0.005	0.008 ±0.010	-0.001 ±0.001
	P value	0.08	0.28	0.31	0.46	0.30

^a^Mixed-effects linear regression model adjusted with spherical equivalent refraction, intraocular pressure and central corneal thickness.

^b^Statistically significant

Twenty-five participants (14 females, 11 males) with a mean age of 64.76 ±3.45 years (range, 60–74 years) were in the LT group and 56 participants (39 females, 17 males) with a mean age of 66.38 ±3.33 years (range, 61–77 years) were in the NT group. Demographic data in each group were shown in Table 2. There was no difference in corneal biomechanical parameters between the LT and NT groups (Table 3).

**Table 2:** Demographic data of the low and normal tear production groups.

Demographic data	Low tear production	Normal tear production	P value^a^
Median (IQR)	(n = 25)	(n = 56)	
OSDI scores	10.42 (4.17, 16.67)	17.08 (6.53, 21.53)	0.27
Tear film break-up time (seconds)	5 (3.67, 5.67)	5.33 (3.33, 8.33)	0.48
Conjunctival staining scores	0 (0, 2)	0 (0, 1.5)	0.77
Corneal staining scores	0 (0, 1)	0 (0, 1)	0.64
Schirmer test (mm)	7 (4, 9)	20 (15, 27.5)	<0.01^b^
Spherical equivalent refraction (diopter)	1.38 (-0.62, 1.81)	1.06 (0, 2)	0.85
Intraocular pressure (mmHg)	12 (10, 14)	14 (11.5, 15)	0.06
Central corneal thickness (μm)	550 (530, 573)	543 (527, 572)	0.91

^a^Mann Whitney U-test

^b^Statistically significant

**Table 3:** Corneal biomechanical parameters of the low and normal tear production groups.

Corneal biomechanical parameters	Low tear production	Normal tear production	P value^a^
Mean ±SD (95% CI)	(n = 25)	(n = 56)	
First applanation time (A1T)	6.74±0.06 (6.63 to 6.85)	6.78±0.03 (6.71 to 6.85)	0.58
Second applanation time (A2T)	21.62±0.08 (21.46 to 21.77)	21.55±0.05 (21.45 to 21.64)	0.44
First applanation length (A1L)	2.22±0.07 (2.08 to 2.37)	2.25±0.04 (2.16 to 2.34)	0.78
Second applanation length (A2L)	1.81±0.08 (1.66 to 1.97)	1.90±0.05 (1.81 to 2.00)	0.33
First applanation velocity (A1V)	0.16±0.00 (0.15 to 0.17)	0.15±0.00 (0.15 to 0.16)	0.10
Second applanation velocity (A2V)	-0.30±0.01 (-0.31 to -0.29)	-0.30±0.00 (-0.30 to -0.29)	0.45
Highest concavity time (HC-time)	16.83±0.10 (16.62 to 17.04)	16.99±0.06 (16.86 to 17.12)	0.19
Highest concavity peak distance (HC-PD)	5.21±0.05 (5.10 to 5.31)	5.17±0.03 (5.11 to 5.23)	0.53
Highest concavity radius (HC-radius)	7.31±0.17 (6.97 to 7.66)	7.09±0.11 (6.88 to 7.30)	0.28
Highest concavity deformation amplitude (HC-DA)	1.18±0.02 (1.14 to 1.22)	1.19±0.01 (1.17 to 1.22)	0.53

^a^ANCOVA test adjusted with spherical equivalent refraction, intraocular pressure and central corneal thickness.

In the LT group, OSDI scores were positively associated with HC-time (P<0.01) and HC-DA (P = 0.04). Moreover, conjunctival staining scores showed significant negative associations with A2L (P = 0.01) and A2V (P<0.01), and significant positive association with HC-PD (P = 0.04). There were no associations between TBUT, corneal staining scores and Schirmer test with corneal biomechanical parameters (Table 4).

**Table 4:** The associations between symptoms and signs of dry eye disease and corneal biomechanical parameters in the low tear production group.

Corneal biomechanical parameters		Symptoms and signs of dry eye disease^a^				
		OSDI scores	Tear film break-up time	Conjunctival staining scores	Corneal staining scores	Schirmer test
First applanation time (A1T)	Coefficient ±SE	-0.002 ±0.002	0.020 ±0.019	-0.019 ±0.015	-0.029 ±0.033	-0.008 ±0.012
	P value	0.37	0.29	0.21	0.39	0.52
Second applanation time (A2T)	Coefficient ±SE	0.002 ±0.003	-0.037 ±0.034	0.045 ±0.026	-0.016 ±0.059	-0.003 ±0.022
	P value	0.52	0.28	0.08	0.79	0.88
First applanation length (A1L)	Coefficient ±SE	0.003 ±0.003	-0.001 ±0.034	0.019 ±0.027	-0.023 ±0.058	-0.033 ±0.021
	P value	0.41	0.97	0.47	0.70	0.11
Second applanation length (A2L)	Coefficient ±SE	0.002 ±0.004	0.012 ±0.036	-0.063 ±0.025	0.027 ±0.059	0.009 ±0.022
	P value	0.54	0.73	**0.01**^**b**^	0.65	0.70
First applanation velocity (A1V)	Coefficient ±SE	0.000 ±0.000	0.000 ±0.002	0.000 ±0.002	-0.002 ±0.004	0.000 ±0.001
	P value	0.94	0.90	0.97	0.48	0.71
Second applanation velocity (A2V)	Coefficient ±SE	0.000 ±0.000	0.003 ±0.003	-0.006 ±0.002	0.003 ±0.004	0.002 ±0.002
	P value	0.08	0.28	**<0.01**^**b**^	0.51	0.22
Highest concavity time (HC-time)	Coefficient ±SE	0.013 ±0.004	0.034 ±0.051	-0.073 ±0.039	-0.052 ±0.089	-0.015 ±0.033
	P value	**<0.01**^**b**^	0.51	0.06	0.56	0.65
Highest concavity peak distance (HC-PD)	Coefficient ±SE	0.001 ±0.002	0.017 ±0.023	0.036 ±0.018	0.031 ±0.040	-0.011 ±0.015
	P value	0.62	0.48	**0.04**^**b**^	0.45	0.46
Highest concavity radius (HC-radius)	Coefficient ±SE	-0.012 ±0.007	0.112 ±0.072	0.047 ±0.060	0.097 ±0.128	-0.014 ±0.049
	P value	0.08	0.12	0.43	0.45	0.78
Highest concavity deformation amplitude (HC-DA)	Coefficient ±SE	0.002 ±0.001	-0.013 ±0.011	0.009 ±0.009	0.011 ±0.019	-0.002 ±0.007
	P value	**0.04**^**b**^	0.20	0.31	0.56	0.73

^a^Mixed-effects linear regression model adjusted with spherical equivalent refraction, intraocular pressure and central corneal thickness.

^b^Statistically significant

In the NT group, Schirmer test was found to be positively associated with A1T (P = 0.02) and negatively associated with HC-DA (P = 0.03). Also, there was positive association between corneal staining scores and A2L (P<0.01). There were no associations between OSDI, TBUT and conjunctival staining scores with corneal biomechanical parameters (Table 5).

**Table 5:** The associations between symptoms and signs of dry eye disease and corneal biomechanical parameters in the normal tear production group.

Corneal biomechanical parameters		Symptoms and signs of dry eye disease^a^				
		OSDI scores	Tear film break-up time	Conjunctival staining scores	Corneal staining scores	Schirmer test
First applanation time (A1T)	Coefficient ±SE	0.001 ±0.003	0.021 ±0.014	0.004 ±0.019	0.012 ±0.039	0.010 ±0.004
	P value	0.86	0.13	0.85	0.77	**0.02**^**b**^
Second applanation time (A2T)	Coefficient ±SE	-0.006 ±0.004	0.001 ±0.018	0.000 ±0.023	-0.006 ±0.048	-0.003 ±0.005
	P value	0.11	0.95	0.99	0.91	0.55
First applanation length (A1L)	Coefficient ±SE	-0.002 ±0.004	-0.008 ±0.017	0.027 ±0.022	0.032 ±0.046	0.005 ±0.005
	P value	0.67	0.63	0.22	0.48	0.37
Second applanation length (A2L)	Coefficient ±SE	0.003 ±0.004	-0.005 ±0.018	0.006 ±0.024	0.133 ±0.046	0.005 ±0.005
	P value	0.36	0.80	0.80	**<0.01**^**b**^	0.35
First applanation velocity (A1V)	Coefficient ±SE	0.000 ±0.000	0.000 ±0.001	0.000 ±0.001	0.000 ±0.002	0.000 ±0.000
	P value	0.86	0.56	0.75	0.87	0.16
Second applanation velocity (A2V)	Coefficient ±SE	0.000 ±0.000	0.000 ±0.002	-0.001 ±0.002	0.005 ±0.004	0.000 ±0.000
	P value	0.19	0.78	0.61	0.28	0.48
Highest concavity time (HC-time)	Coefficient ±SE	0.002 ±0.005	0.041 ±0.022	0.004 ±0.030	-0.051 ±0.061	0.011 ±0.007
	P value	0.75	0.06	0.89	0.41	0.09
Highest concavity peak distance (HC-PD)	Coefficient ±SE	-0.005 ±0.003	-0.007 ±0.012	0.012 ±0.016	-0.006 ±0.033	-0.006 ±0.004
	P value	0.05	0.56	0.45	0.85	0.08
Highest concavity radius (HC-radius)	Coefficient ±SE	-0.014 ±0.008	0.032 ±0.040	-0.016 ±0.054	-0.044 ±0.110	0.007 ±0.012
	P value	0.10	0.43	0.76	0.69	0.59
Highest concavity deformation amplitude (HC-DA)	Coefficient ±SE	0.001 ±0.001	-0.002 ±0.005	0.003 ±0.006	0.005 ±0.012	-0.003 ±0.001
	P value	0.58	0.59	0.61	0.68	**0.03**^**b**^

^a^Mixed-effects linear regression model adjusted with spherical equivalent refraction, intraocular pressure and central corneal thickness

^b^Statistically significant

Associations between symptoms and signs of DED with the alteration of corneal biomechanical properties were found in this study. Among ten corneal biomechanical parameters detected by Corvis ST, eight parameters were associated with either symptoms or signs of DED. The increase in severity of DED was associated with less stiffness of cornea in both low and normal tear production. Moreover, there was no difference in corneal biomechanics between participants with low and normal tear production.

Proper assessment of corneal biomechanical parameters is necessary since they are associated with diagnosis and management of various ophthalmic diseases including preoperative screening of corneal refractive surgery candidates and the precise intraocular pressure measurement and interpretation. The conditions associated with the alteration of corneal biomechanics have previously been mentioned including keratoconus, post-corneal refractive surgery, corneal edema, corneal scar, autoimmune diseases, myopia, and glaucoma [14–18]. In the current study, we found that DED, a common ocular surface disease, was significantly associated with the alteration of corneal biomechanics.

Multiple studies have shown that there was no association between symptoms and signs of DED [19, 20]. However, we found that both symptoms and signs of DED except TBUT were significantly correlated with at least one among ten corneal biomechanical parameters. After an air impulse generated by Corvis ST, the patients with more severe dry eye symptoms showed smaller concavity radius (HC-radius). The patients with higher conjunctival staining scores demonstrated lower second applanation velocity (A2V). The patients with higher corneal staining scores exhibited longer length of flattened cornea at second applanation (A2L) and the patients with lower tear production evaluated by Schirmer test displayed a higher speed of corneal apex at first applanation (A1V) and shorter first applanation time (A1T). Interestingly, the alteration of corneal biomechanical parameters in patients with symptoms and signs of DED except A2L were consistent with the alteration that was found in patients with keratoconus [21–26].

Theoretically, an earlier applanation, which is represented as a shorter time to the first applanation (A1T) and a higher velocity at the first applanation (A1V), suggests a softer cornea. In addition, an increase in HC-DA and a decrease in HC-radius means a greater change in corneal shape after an air impulse, which can be interpreted as reduced corneal stiffness. Likewise, a higher velocity at the second applanation (A2V) suggested more compliant cornea. In clinical setting, various studies have compared corneal biomechanical parameters between normal and keratoconic eyes using Corvis ST. Five corneal biomechanical parameters including A1T, A2T, A1V, A2V, HC-radius and HC-DA consistently showed the alteration between normal and keratoconic eyes, while the other parameters demonstrated no significant difference [21–26]. Similar to keratoconic eyes, we found that the increase in severity of DED resulted in the alteration of corneal biomechanical parameters including HC-radius, A2V and A1T. Thus, our results indicated that the corneas of patients with DED became weaker and more deformable. The increase in severity of DED resulted in the more compliant cornea.

No difference in corneal biomechanics between low and normal tear production was detected. Both participants with low and normal tear production showed the associations between either symptoms or signs of DED and the corneal biomechanical parameters. In participants with low tear production, the increase in OSDI scores and conjunctival staining scores were associated with more compliant cornea. Furthermore, the lower Schirmer test was associated with the more compliant cornea in participants with normal tear production.

There were a few studies which investigated corneal biomechanics in patients with DED. Most studies have shown that patients with autoimmune-mediated DED including Sjogren’s syndrome and ocular graft versus host disease had more compliant corneas compared with healthy corneas [27–29]. Long Q et al. evaluated corneal biomechanics in dry eye patients without systemic autoimmune diseases using Corvis ST and found that the time to reach the highest concavity of cornea (HC-time) was significantly reduced in patients with DED compared with normal [30]. In contrast, Firat PG and Doganay S. demonstrated no difference in corneal biomechanics between patients with and without DED using Ocular Response Analyzer [31]. Compared to our study, the previous studies included smaller sample sizes and did not adjust the biomechanical parameters with intraocular pressure, spherical equivalent refraction, and corneal thickness, which are considered important factors affecting the biomechanical properties. Most of our patients had no known underlying diseases which could affect corneal biomechanics, except one patient with rheumatoid arthritis.

The alteration in corneal biomechanics in DED could be due to various changes in corneal structure including destruction and apoptosis of corneal epithelium, decrease in corneal subbasal nerve density, inflammation of corneal stroma and decrease corneal endothelial cell density [2]. Corneal stroma, which comprised 90% of total corneal thickness, was the major part that contributed to the corneal biomechanical properties [16]. Various conditions associated with corneal stromal inflammation such as keratoconus, Sjogren syndrome, herpes stromal keratitis have shown to be associated with more compliant corneas [32–36]. Despite that the immunopathogenesis of DED has not yet been fully understood, numerous studies have concluded that ocular surface inflammation displayed a critical role [37–39]. Elevation of inflammatory cytokines, matrix metalloproteinases-9 (MMP-9), chemokines, and infiltration of immune cells have been found in DED [40–42]. MMP-9, which was also found to be elevated in corneal ectatic diseases, including keratoconus and post-LASIK ectasia [43, 44], is a primary enzyme of the ocular surface that causes degradation of the collagen and the protein in extracellular matrix [13]. In addition, Giannaccare G et al. reported that the tear level of MMP-9 was negatively correlated with the value of corneal hysteresis in ocular graft versus host disease patients with DED [27]. Therefore, we believe that the corneal inflammation leads to a more compliant cornea in DED.

Ocular surface inflammation in DED can both cause and be the consequence of corneal and conjunctival epithelial cell damage [39]. Elsheikh A et al. demonstrated that corneal epithelial integrity was responsible for the stability of corneal biomechanics [45]. Thus, the ocular surface damage would further lead to more compliant cornea in DED. Until now, the role of the corneal nerve and corneal endothelium in biomechanics is still unclear. In addition to DED, the findings of decreased corneal nerve density, decreased corneal endothelium cell density, and poor corneal biomechanics, were also found in keratoconus and herpetic stromal keratitis [33, 46]. Moreover, Parissi M. et al. have showed after strengthening cornea by collagen crosslinking treatment, corneal nerve density increased [47]. However, the findings of the decrease in corneal nerve and corneal endothelial cell density as well as the alteration of corneal biomechanics could be a consequence of corneal inflammation, which is normally found in those conditions. The direct contribution of corneal nerve and corneal endothelial cells on biomechanics need to be investigated in the future.

The limitations of this study are as follows. Firstly, we have adjusted biomechanical parameters with only spherical equivalent refraction, intraocular pressure, and corneal thickness. However, we did not assess all ocular intrinsic factors that may alter corneal biomechanics, such as axial length. Secondly, participants using artificial tears had not been excluded. This may affect the results since biomechanical properties can be altered by corneal hydration. Finally, the cause-effect relationship between symptoms and signs of DED with corneal biomechanics cannot be ascertained.

## Conclusions

In conclusion, the current study showed that patients with DED had more compliant cornea. An increase in severity of DED was associated with the worsening of corneal biomechanics in both patients with low and normal tear production. The corneal biomechanics might serve as new parameters for categorizing dry eye severity.

## Supporting information

S1 File The data spreadsheet. (XLSX)
